# Is lobectomy superior to sub-lobectomy in non-small cell lung cancer with pleural invasion? A population-based competing risk analysis

**DOI:** 10.1186/s12885-022-09634-w

**Published:** 2022-05-13

**Authors:** Xue Song, Yangyang Xie, Yurou Zhu, Yafang Lou

**Affiliations:** 1 Department of Respiratory and Critical Care Medicine, Hangzhou TCM Hospital Affiliated to Zhejiang Chinese Medical University, #453, Tiyuchang Road, Xihu District, Hangzhou, 310000 Zhejiang province China; 2 Department of General Surgery, Hangzhou TCM Hospital Affiliated to Zhejiang Chinese Medical University, #453, Tiyuchang Road, Xihu District, Hangzhou, 310000 Zhejiang province China

**Keywords:** Non-small cell lung cancer, Lobectomy, Sub-lobectomy, Pleural invasion, SEER Program

## Abstract

**Background:**

Pleural invasion (PL) has been regarded as an unfavorable prognostic factor for non-small cell lung cancer (NSCLC). But there was no agreement on the optimal surgical extent in NSCLC patients with PL. We aimed to compare the survival outcomes of lobectomy and sub-lobectomy in these patients.

**Method:**

2717 patients were included in the Surveillance, Epidemiology, and End Results (SEER) database and divided into the lobectomy and sub-lobectomy groups. The propensity score matching (PSM) and competing risk analysis were implemented. Then the predictive nomogram was constructed and validated.

**Results:**

2230 Patients received lobectomy while the other 487 patients underwent sub-lobectomy. After 1:1 PSM, the cumulative incidence of cancer-specific death (CSD) was lower in the lobectomy group compared with the sub-lobectomy group (1-year: 12% vs. 15%; 3-year: 30% vs. 37%, 5-year: 34% vs. 45%, *P* = 0.04). According to the subgroup analysis, the patients who underwent lobectomy suffered lower CSD in the N0–1 stage, adenocarcinoma, and PL-2 cohort (*p* < 0.05). And there was a significant relationship between the sub-lobectomy group and CSD in the multivariate competing risks regression analysis (HR, 1.26; 95%CI, 1.02–1.56; *P* = 0.034). Furthermore, a competing event nomogram was constructed to assess the 1-, 3-, and 5-year chances of CSD based on the variables from the multivariate analysis. The 1-, 3-, 5-year area under the receiver operating characteristic curve (AUC) values were 0.720, 0.706, and 0.708 in the training cohort, and 0.738, 0.696, 0.680 in the validation cohorts, respectively. And calibration curves demonstrated ideal consistency between the predicted and observed probabilities of CSD.

**Conclusion:**

Lobectomy should be considered the preferred surgery compared to sub-lobectomy for NSCLC patients with PL. The proposed nomograms presented great prediction ability for these patients.

**Supplementary Information:**

The online version contains supplementary material available at 10.1186/s12885-022-09634-w.

## Introduction

Lung cancer is the leading cause of cancer mortality among malignant tumors [1]. Among the common subtypes of lung cancer, non-small cell lung cancer (NSCLC) represents approximately 85% of the overall patients [2], with a 5-year relative survival rate to be 12–15% [3]. Pleural invasion (PL), defined as tumor invasion beyond the elastic layer, has been identified as an independent pathological feature associated with more aggressive biological behavior [4, 5]. Previous studies demonstrated that NSCLC patients with PL suffered a higher incidence of poor tumor differentiation, mediastinal lymph node metastatic spread, postoperative recurrence, and poor survival [6–9].

Lobectomy (Lob) and sub-lobectomy (Sub-lob), the most commonly adopted surgical methods, are the preferred treatment for lung cancer [10]. Lob and systematic lymph node dissection are the gold standard treatment modalities for early-stage NSCLC, which provide more aggressive and comprehensive excision. And Sub-lob presents superiority in patients with significant comorbidities or limited pulmonary function which may be technically easier and carry fewer perioperative complications [11]. As one of the essential elements regarding NSCLC staging, PL directly affects the surgical strategies and prognosis judgment in lung cancer [12]. However, there is no agreement on the optimal surgical extent for NSCLC with PL. Wo et al. [13] and Yu et al. [14] analyzed the prognostic value of surgical extent in NSCLC patients with PL based on the SEER database, indicating that patients who underwent Sub-lob had shorter survival times than those who underwent Lob. Conversely, Moon et al. [15] investigated the surgical outcomes of 271 NSCLC patients with PL and revealed that survival rate did not differ significantly by surgical extent. Thus, controversy still exists regarding the better surgery type (Lob or Sub-lob) in these patients.

Hence, this retrospective study aimed to use the Surveillance, Epidemiology, and End Results (SEER) database to compare the survival outcomes of Lob and Sub-lob in NSCLC patients with PL and construct a predictive nomogram.

## Materials and methods

### Data source and patient selection

Patients were extracted from the SEER 18 regions database [Incidence-SEER Research Plus data, 18 Registries, Nov 2000 Sub (2000–2018)] using SEER*Sat software (Version 8.3.5). Patients who met the inclusion criteria were identified: (1) age over 18 years; (2) patients were diagnosed according to the International Classification of Disease histology code for Oncology (ICD-0-3) with adenocarcinoma (8140-8147, 8255, 8260, 8310,8323, 8480, 8481, 8490,8550,8572), squamous cell carcinoma (8050-8052, 8070-8078), and other pathologies including large-cell carcinoma (8012-8014), undifferentiated tumors (8020-8022) and carcinomas not otherwise specified (8010); (3) patients underwent surgical resection of lung cancer: Lob and Sub-lob (segmentectomy and wedge resection); (4) patients with exact pathologic pleural invasion status of PL1, PL2 and PL3.

**Fig. 1:**
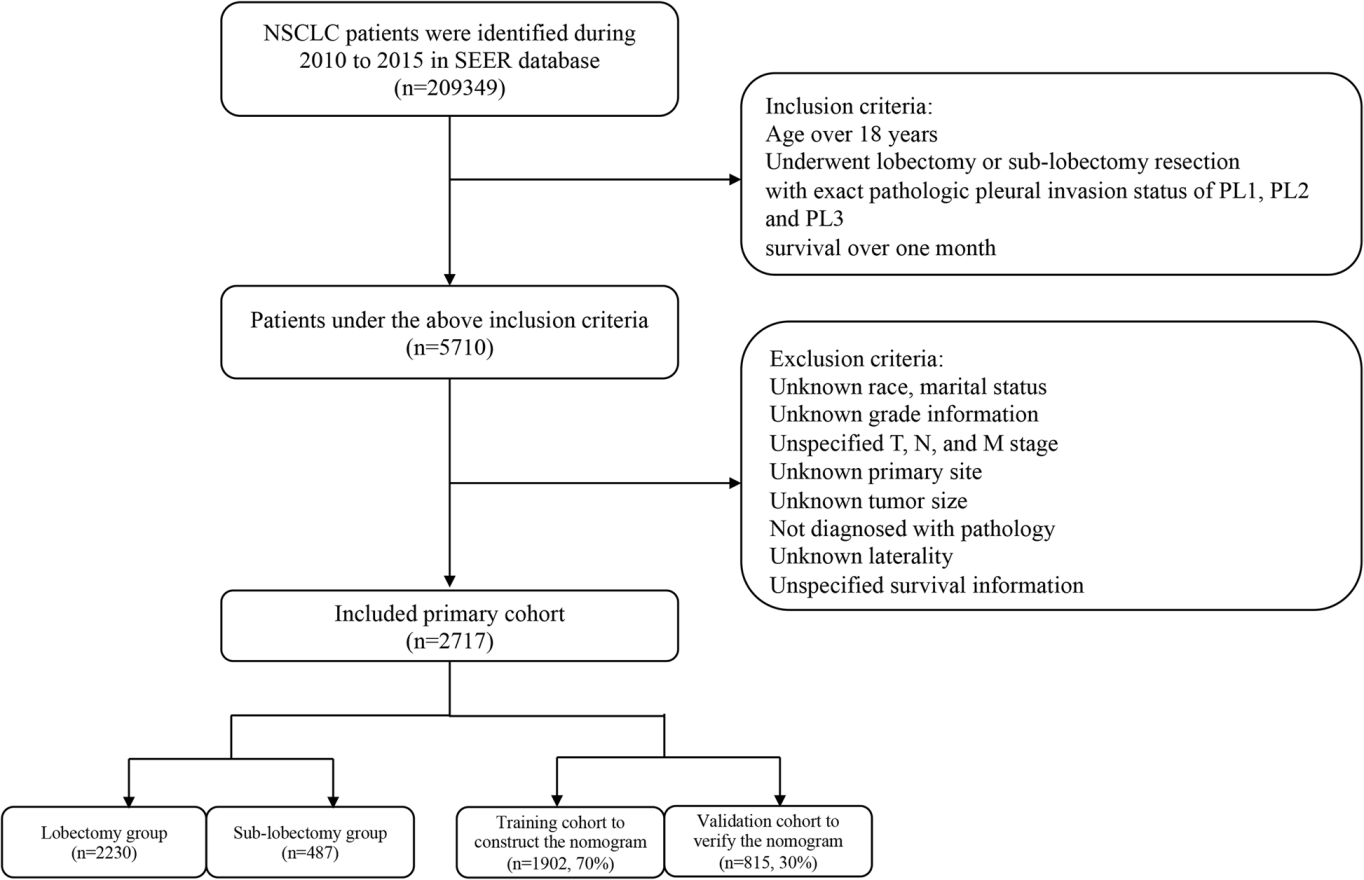
The workflow of the patient selection process

The exclusion criteria were: (1) patients who survived less than 1 month; (2) more than one malignancy; (3) patients with incomplete demographic, clinic-pathological, treatment, and follow-up information. Ultimately, 2717 cases were included in the study. All patients were first divided into Lob and Sub-lob cohorts to perform competing risk analyses. Then the same population was split into training and validation groups to construct a predictive nomogram. The detailed patient selection workflow is shown in Fig. 1.

### Clinicopathological variables

Demographic data (year of diagnosis, age, gender, race, marital status), grade, T stage, N stage, metastasis, pathology, pleural invasion, primary site, laterality, tumor size, radiation, chemotherapy, and prognostic information were retrieved from the SEER data repository. The NSCLC patients were reclassified according to the 8th edition TNM classification based on the 7th edition recorded in the SEER database. PL status was obtained from the variables of collaborative stage site-specific factor 2 (2004+) for lung cancer: tumor invasion beyond the visceral elastic layer (PL-1); tumor invasion to the visceral layer (PL-2); tumor extends to the parietal pleura (PL-3). PL-0 and unspecified pleural invasion were excluded based on the research purpose.

### Statistical analysis

Continuous variables were expressed as means and standard deviations, and categorical data were summarized as frequency counts and percentages. Differences in the baseline clinicopathological variables were tested using t-test and chi-square test.

All patients were split into Lob and Sub-lob cohorts in the competing risk analyses. OCD (other causes of death) was regarded as an event competing with CSD (cancer-specific death). The endpoints of interest were divided into alive, CSD, and OCD. The cumulative incidence function (CIF) was used for univariate analyses, then the intergroup difference in the CIF was identified by Gray’s test. For the multivariate analysis, Fine and Gray’s proportional subdistribution hazard model was further used to determine the prognostic factors with the R package “cmprsk” [16].

Using a one-to-one nearest-neighbor algorithm, the propensity score matching (PSM) method was a novel statistical method that could minimize the heterogeneity and mimic randomized controlled trials [17]. We used standardized difference (SD) to present the change of variables before and after PSM. SD ≤0.1 indicated ideal balances in the baseline parameters [18].

Then the overall patients were randomly divided into a training group (70%, *n* = 1902) and a validation group (30%, *n* = 815). The prognostic factors identified in the competing risk model were applied to construct a 1-, 3-, and 5-year CSD nomogram in the training dataset. The detailed process was based on the step-by-step method provided by Zhang et al. [19]. The performance of the nomogram was first tested in the training group and subsequently in the validation group by the area under the receiver operating characteristic curve (AUC) values and calibration curves. 1000 bootstrap resamples were used to analyze the expected and observed survival probabilities in the calibration curves. The receiver operating characteristic (ROC) curves were also shown to highlight the built model’s prediction power and calculate AUC.

All statistical analyses and visualization were based on R software (version 4.0.3, The R Foundation for Statistical Computing, Vienna, Austria; http://www.r-project.org). A two-tailed *P* < 0.05 was indicated to be statistically significant.

## Results

### Clinicopathological characteristics

2717 patients were finally recruited in the SEER database from 2010 to 2015. Of the overall patients, 2230 (82.08%) and 487 (17.92%) underwent Lob and Sub-lob, respectively. There were significant dissimilarities among the two cohorts in the characteristics, including age, marital status, laterality, T stage, N stage, metastasis, tumor size, radiation, and chemotherapy (all *p* < 0.05). The patients underwent Lob tended to present higher proportion of married status (60.0% vs. 53.2%), T3 stage (33.3% vs. 21.6%), N1 stage (16.5% vs. 4.1%), N2 stage (16.6 vs. 12.9%), M0 stage (95.9% vs. 83.8%), chemotherapy (40.4% vs. 31.8%). The Sub-lob group presented high percentage in T2 stage (64.9% vs. 62.2%), T4 stage (13.6% vs. 4.6%) and radiotherapy (22.6% vs. 16.9%).

Given unmatched parameters between the two cohorts, we performed 1:1 PSM to reduce the influence of potential confounders. After PSM, SD in most variables was less than 0.1, which indicated good balancing performance (Fig. S1). Ultimately, 856 patients were separated into the Lob group (*n* = 428) and Sub-lob group (*n* = 428). The baseline characteristics before and after PSM are presented in Table 1.

**Table 1:** The descriptive characteristics of NSCLC patients with PL before and after PSM

Characteristics	Before PSM			*P* value	After PSM			*P* value
	All	Lobectomy	Sub-lobectomy		All	Lobectomy	Sub-lobectomy	
	*N* = 2717	*N* = 2230	*N* = 487		*N* = 856	*N* = 428	*N* = 428	
Year at diagnosis				0.125				0.945
2010–2012	1273 (46.9%)	1029 (46.1%)	244 (50.1%)		418 (48.8%)	210 (49.1%)	208 (48.6%)	
2013–2015	1444 (53.1%)	1201 (53.9%)	243 (49.9%)		438 (51.2%)	218 (50.9%)	220 (51.4%)	
Age	69.0 (14.1)	68.0 (13.8)	72.0 (15.6)	< 0.001	71.0 (13.3)	71.0 (16.6)	71.0 (16.1)	0.258
Gender				0.539				0.632
Female	1380 (50.8%)	1126 (50.5%)	254 (52.2%)		446 (52.1%)	219 (51.2%)	227 (53.0%)	
Male	1337 (49.2%)	1104 (49.5%)	233 (47.8%)		410 (47.9%)	209 (48.8%)	201 (47.0%)	
Race				0.914				0.235
White	2185 (80.4%)	1792 (80.4%)	393 (80.7%)		712 (83.2%)	363 (84.8%)	349 (81.5%)	
Non-White	532 (19.6%)	438 (19.6%)	94 (19.3%)		144 (16.8%)	65 (15.2%)	79 (18.5%)	
Marital status				0.006				0.336
Married	1598 (58.8%)	1339 (60.0%)	259 (53.2%)		473 (55.3%)	244 (57.0%)	229 (53.5%)	
Unmarried	1119 (41.2%)	891 (40.0%)	228 (46.8%)		383 (44.7%)	184 (43.0%)	199 (46.5%)	
Grade				0.85				0.841
I	218 (8.0%)	176 (7.9%)	42 (8.6%)		65 (7.6%)	29 (6.8%)	36 (8.4%)	
II	1349 (49.7%)	1114 (50.0%)	235 (48.3%)		424 (49.5%)	213 (49.8%)	211 (49.3%)	
III	1112 (40.9%)	910 (40.8%)	202 (41.5%)		357 (41.7%)	181 (42.3%)	176 (41.1%)	
IV	38 (1.4%)	30 (1.3%)	8 (1.6%)		10 (1.2%)	5 (1.2%)	5 (1.2%)	
T stage				< 0.001				0.712
T2	1702 (62.6%)	1386 (62.2%)	316 (64.9%)		588 (68.7%)	289 (67.5%)	299 (69.9%)	
T3	847 (31.2%)	742 (33.3%)	105 (21.6%)		196 (22.9%)	103 (24.1%)	93 (21.7%)	
T4	168 (6.2%)	102 (4.6%)	66 (13.6%)		72 (8.4%)	36 (8.4%)	36 (8.4%)	
N stage				< 0.001				0.481
N0	1883 (69.3%)	1486 (66.6%)	397 (81.5%)		691 (80.7%)	340 (79.4%)	351 (82.0%)	
N1	387 (14.2%)	367 (16.5%)	20 (4.1%)		48 (5.6%)	29 (6.8%)	19 (4.4%)	
N2	433 (15.9%)	370 (16.6%)	63 (12.9%)		110 (12.9%)	55 (12.9%)	55 (12.9%)	
N3	14 (0.5%)	7 (0.3%)	7 (1.4%)		7 (0.8%)	4 (0.9%)	3 (0.7%)	
Metastasis				< 0.001				0.824
M0	2547 (93.7%)	2139 (95.9%)	408 (83.8%)		765 (89.4%)	381 (89.0%)	384 (89.7%)	
M1	170 (6.3%)	91 (4.1%)	79 (16.2%)		91 (10.6%)	47 (11.0%)	44 (10.3%)	
Pathology				0.915				0.849
Adenocarcinoma	1978 (72.8%)	1625 (72.9%)	353 (72.5%)		618 (72.2%)	308 (72.0%)	310 (72.4%)	
Others	67 (2.5%)	56 (2.5%)	11 (2.3%)		16 (1.9%)	7 (1.6%)	9 (2.1%)	
Squamous cell carcinoma	672 (24.7%)	549 (24.6%)	123 (25.3%)		222 (25.9%)	113 (26.4%)	109 (25.5%)	
Pleural invasion				0.073				0.645
PL-1	1280 (47.1%)	1073 (48.1%)	207 (42.5%)		389 (45.4%)	201 (47.0%)	188 (43.9%)	
PL-2	1076 (39.6%)	869 (39.0%)	207 (42.5%)		347 (40.5%)	170 (39.7%)	177 (41.4%)	
PL-3	361 (13.3%)	288 (12.9%)	73 (15.0%)		120 (14.0%)	57 (13.3%)	63 (14.7%)	
Primary site				0.598				0.846
Lower lobe	802 (29.5%)	667 (29.9%)	135 (27.7%)		243 (28.4%)	125 (29.2%)	118 (27.6%)	
Others	219 (8.1%)	177 (7.9%)	42 (8.6%)		73 (8.5%)	37 (8.6%)	36 (8.4%)	
Upper lobe	1696 (62.4%)	1386 (62.2%)	310 (63.7%)		540 (63.1%)	266 (62.1%)	274 (64.0%)	
Laterality				< 0.001				0.632
Left	1108 (40.8%)	868 (38.9%)	240 (49.3%)		406 (47.4%)	207 (48.4%)	199 (46.5%)	
Right	1609 (59.2%)	1362 (61.1%)	247 (50.7%)		450 (52.6%)	221 (51.6%)	229 (53.5%)	
Tumor size	3.0 (1.0)	3.2 (1.1)	2.2 (0.7)	< 0.001	2.4 (0.9)	2.5 (0.8)	2.3 (1.1)	0.04
Radiation				0.003				0.675
None	2231 (82.1%)	1854 (83.1%)	377 (77.4%)		676 (79.0%)	335 (78.3%)	341 (79.7%)	
Radiotherapy	486 (17.9%)	376 (16.9%)	110 (22.6%)		180 (21.0%)	93 (21.7%)	87 (20.3%)	
Chemotherapy				< 0.001				0.504
None	1660 (61.1%)	1328 (59.6%)	332 (68.2%)		596 (69.6%)	293 (68.5%)	303 (70.8%)	
Chemotherapy	1057 (38.9%)	902 (40.4%)	155 (31.8%)		260 (30.4%)	135 (31.5%)	125 (29.2%)	

### Survival analysis

Cumulative incidence plots were constructed considering the competing risk factors, presenting significantly lower CSD in the Lob group (*P* < 0.001). And the patients in the Lob group suffered lower 1-, 3-, and 5-year CIF of CSD than the patients underwent Sub-lob (1-year: 12% vs. 17%; 3-year: 29% vs. 39%, 5-year: 39% vs. 47%, P < 0.001) (Table 2). Subsequently, the subgroups analysis for T stage, N stage, metastasis, pathology, and pleural invasion extent were performed. The results showed that the patients who underwent Lob suffered lower CSD in the T2–3 stage, N0–1 stage, adenocarcinoma, and PL-2 cohort (Fig. S2). In the multivariable competing risks regression analysis, a significant correlation was found between the Sub-lob group and CSD (HR, 1.27; 95%CI, 1.08–1.51; *p* = 0.004).

**Table 2:** The cumulative incidence of CSD and OCD in two cohorts before and after PSM

	Cancer-specific death (%)			*P* value	Other causes death (%)			*P* Value
Before PSM	1-year CIF	3-year CIF	5-year CIF		1-year CIF	3-year CIF	5-year CIF	
Lobectomy	0.12	0.29	0.39	< 0.01	0.03	0.07	0.10	< 0.01
Sub-lobectomy	0.17	0.39	0.47		0.03	0.11	0.16	
After PSM								
Lobectomy	0.12	0.3	0.34	0.04	0.03	0.07	0.11	0.05
Sub-lobectomy	0.15	0.37	0.45		0.04	0.11	0.17	

*CIF* cumulative incidences function

**Fig. 2:**
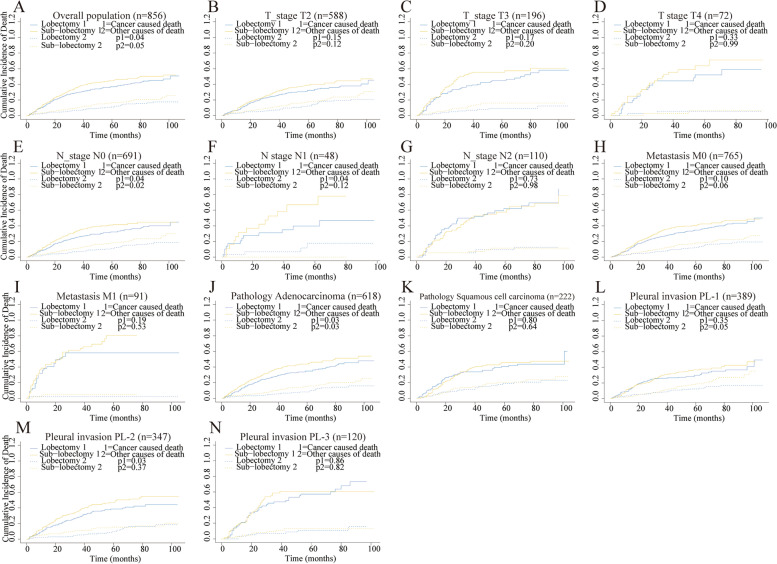
Cumulative incidence curves for the NSCLC patients with PL in overall cases and different subgroups after PSM. Overall patients (**A**), T2 (**B)**, T3 (**C**), T4 (**D**), N0 (**E**), N1 (**F**), N2 (**G**), M0 (**H**), M1 (**I**), adenocarcinoma (**J**), squamous cell carcinoma (**K**), PL-1 (**L**), PL-2 (**M**) and PL-3 (**N**) cohorts

After 1:1 PSM, significant difference was still found for the 1-, 3-, and 5-year CIF of CSD between the two groups (1-year: 12% vs. 15%; 3-year: 30% vs. 37%, 5-year: 34% vs. 45%, *P* = 0.04) (Table 2). Then the subgroups analyses were performed again, indicating that the patients who underwent sub-lobar resection suffered higher CSD in the N0–1 stage, adenocarcinoma, and PL-2 cohort (Fig. 2). There was a significant relationship between the Sub-lob group and CSD in the multivariate competing risks regression analysis (HR, 1.26; 95%CI, 1.02–1.56; *P* = 0.034) (Table 3). And the relationship between the Sub-lob group and OCD was not significant (HR, 1.37; 95%CI, 0.96–1.95, *P* = 0.078) (Table S1).

**Table 3:** The results of the multivariate subdistribution hazards model on CSD before and after PSM

Characteristics	Before PSM			After PSM		
	HR	95%CI	*P* value	HR	95%CI	*P* value
Surgery						
Lobectomy	Reference			Reference		
Sub-lobectomy	1.27	1.08–1.51	0.004	1.26	1.02–1.56	0.034
Age	1.02	1.01–1.03	< 0.001	1.02	1–1.03	0.007
Gender						
Female	Reference			Reference		
Male	1.16	1.03–1.32	0.016	1.21	0.97–1.5	0.091
Race						
White	Reference			Reference		
Non-White	0.94	0.81–1.1	0.451	0.86	0.64–1.17	0.350
Marital status						
Married	Reference			Reference		
Unmarried	1	0.88–1.13	0.992	1.06	0.85–1.32	0.601
Grade						
I	Reference			Reference		
II	1.43	1.09–1.87	0.011	1.28	0.79–2.07	0.311
III	1.71	1.3–2.25	< 0.001	1.75	1.08–2.83	0.023
IV	2.52	1.5–4.26	0.001	3.29	1.32–8.21	0.011
T stage						
T2	Reference			Reference		
T3	1.15	0.96–1.39	0.131	1.3	0.9–1.88	0.170
T4	1.14	0.86–1.5	0.362	1.21	0.78–1.89	0.394
N stage						
N0	Reference			Reference		
N1	1.56	1.31–1.87	< 0.001	1.53	0.93–2.51	0.093
N2	2.07	1.75–2.45	< 0.001	1.85	1.36–2.52	< 0.001
N3	1.61	0.74–3.5	0.230	3.37	1.99–5.71	< 0.001
Metastasis						
M0	Reference			Reference		
M1	2.3	1.79–2.95	< 0.001	2.48	1.71–3.59	< 0.001
Pathology						
Adenocarcinoma	Reference			Reference		
Others	1.42	0.93–2.15	0.101	1.68	0.75–3.74	0.216
Squamous cell carcinoma	0.97	0.83–1.13	0.721	1.01	0.78–1.31	0.953
Pleural invasion						
PL-1	Reference			Reference		
PL-2	1.11	0.97–1.27	0.140	1.25	0.98–1.6	0.078
PL-3	1.57	1.28–1.92	< 0.001	1.37	0.91–2.06	0.131
Primary site						
Lower lobe	Reference			Reference		
Others	1.05	0.82–1.34	0.701	1	0.65–1.54	0.991
Upper lobe	0.89	0.78–1.02	0.089	1	0.79–1.28	0.988
Laterality						
Left	Reference			Reference		
Right	0.86	0.76–0.97	0.017	0.87	0.7–1.09	0.233
Tumor size	1.07	1.03–1.11	< 0.001	1.01	0.96–1.06	0.757
Radiation						
None	Reference			Reference		
Radiotherapy	1.35	1.15–1.58	< 0.001	1.55	1.19–2.03	0.001
Chemotherapy						
None	Reference			Reference		
Chemotherapy	0.88	0.75–1.02	0.098	0.81	0.6–1.09	0.176

*HR* Hazard ratio

### Univariate and multivariate analysis

Then we conducted a second independent analysis. Aiming to construct a prognostic model, we randomly divided the overall patients into a training group (70%, *n* = 1902) and a validation group (30%, *n* = 815). There was no significant discrepancy in clinical baselines between the two groups (Table S2). Univariate analyses were used to calculate the 1-, 3-, and 5-year CIF values of CSD in the training cohort. The result revealed that age, gender, grade, T stage, N stage, metastasis, pathology, pleural invasion, primary site, laterality, tumor size, radiation, chemotherapy, and surgery were significantly related to CSD. We did not incorporate tumor size in further analyses because the variable T stage contained tumor size information. Then the significant variables (*P* < 0.1) were further identified by the multivariate assessment of the Fine-Gray proportional subdistribution hazards model. The multivariate competing risk analysis indicated that age, gender, grade, T stage, N stage, metastasis, pleural invasion, surgery, radiation, and chemotherapy were independent predictors affecting CSD in NSCLC patients with PL (Table 4).

**Table 4:** The cumulative incidences and multivariate subdistribution proportional hazards analysis on CSD

Characteristics	Cause-specific death (%)					Subdistribution proportional hazards model		
	1-year CIF	3-year CIF	5-year CIF	Gray’s test	*P* value	HR	95% CI	*P* value
Year at diagnosis				1.31	0.251			
2010–2012	0.13	0.33	0.41					
2013–2015	0.12	0.3	0.41					
Age				83.16	0.011	1.02	0.98–1.03	< 0.001
Gender				7.94	0.005			
Female	0.11	0.28	0.37			Reference		
Male	0.14	0.34	0.43			1.18	0.85–1.36	0.031
Race				1.2	0.273			
White	0.13	0.32	0.41					
Non-White	0.12	0.3	0.39					
Marital status				0.17	0.681			
Married	0.12	0.31	0.41					
Unmarried	0.13	0.31	0.39					
Grade				35.71	< 0.001			
I	0.09	0.23	0.28			Reference		
II	0.09	0.27	0.37			1.37	0.72–1.91	0.062
III	0.17	0.37	0.46			1.64	0.61–2.3	0.004
IV	0.27	0.58	0.63			2.63	0.38–4.9	0.002
T stage				108.79	< 0.001			
T2	0.08	0.23	0.32			Reference		
T3	0.19	0.44	0.53			1.31	0.77–1.63	0.021
T4	0.28	0.5	0.64			1.29	0.78–1.80	0.143
N stage				107.91	< 0.001			
N0	0.1	0.26	0.33			Reference		
N1	0.15	0.37	0.51			1.8	0.55–2.23	< 0.001
N2	0.21	0.49	0.62			2.48	0.40–3.03	< 0.001
N3	0.2	0.6	NA			2.03	0.50–5.19	0.141
Metastasis				78.48	< 0.001			
M0	0.11	0.29	0.38			Reference		
M1	0.38	0.63	0.7			2.62	0.38–3.57	< 0.001
Pathology				10.46	0.005			
Adenocarcinoma	0.1	0.28	0.39			Reference		
Others	0.33	0.49	0.51			1.53	0.65–2.48	0.081
Squamous cell carcinoma	0.17	0.38	0.44			1.02	0.98–1.22	0.872
Pleural invasion				54.91	< 0.001			
PL-1	0.1	0.27	0.34			Reference		
PL-2	0.13	0.31	0.43			1.1	0.91–1.30	0.245
PL-3	0.21	0.48	0.57			1.57	0.64–2.01	< 0.001
Primary site				7.16	0.028			
Lower lobe	0.13	0.35	0.45			Reference		
Others	0.13	0.3	0.39			1.06	0.94–1.42	0.68
Upper lobe	0.12	0.29	0.38			0.87	0.65–2.48	0.108
Laterality:				6.72	0.011			
Left	0.14	0.34	0.43			Reference		
Right	0.12	0.29	0.39			0.87	0.73–1.15	0.061
Tumor size				547.18	< 0.001			
Radiation				44.65	< 0.001			
None	0.11	0.28	0.37			Reference		
Radiotherapy	0.19	0.45	0.57			1.28	0.78–1.56	0.013
Chemotherapy				15.67	< 0.001			
None	0.12	0.28	0.37			Reference		
Chemotherapy	0.14	0.37	0.46			0.77	0.65–1.29	0.009
Surgery				4.45	0.036			
Lobectomy	0.12	0.3	0.39			Reference		
Sub-lobectomy	0.16	0.38	0.45			1.24	0.81–1.51	0.035

*HR* Hazard ratio

### Constructing and verifying the nomogram

**Fig. 3:**
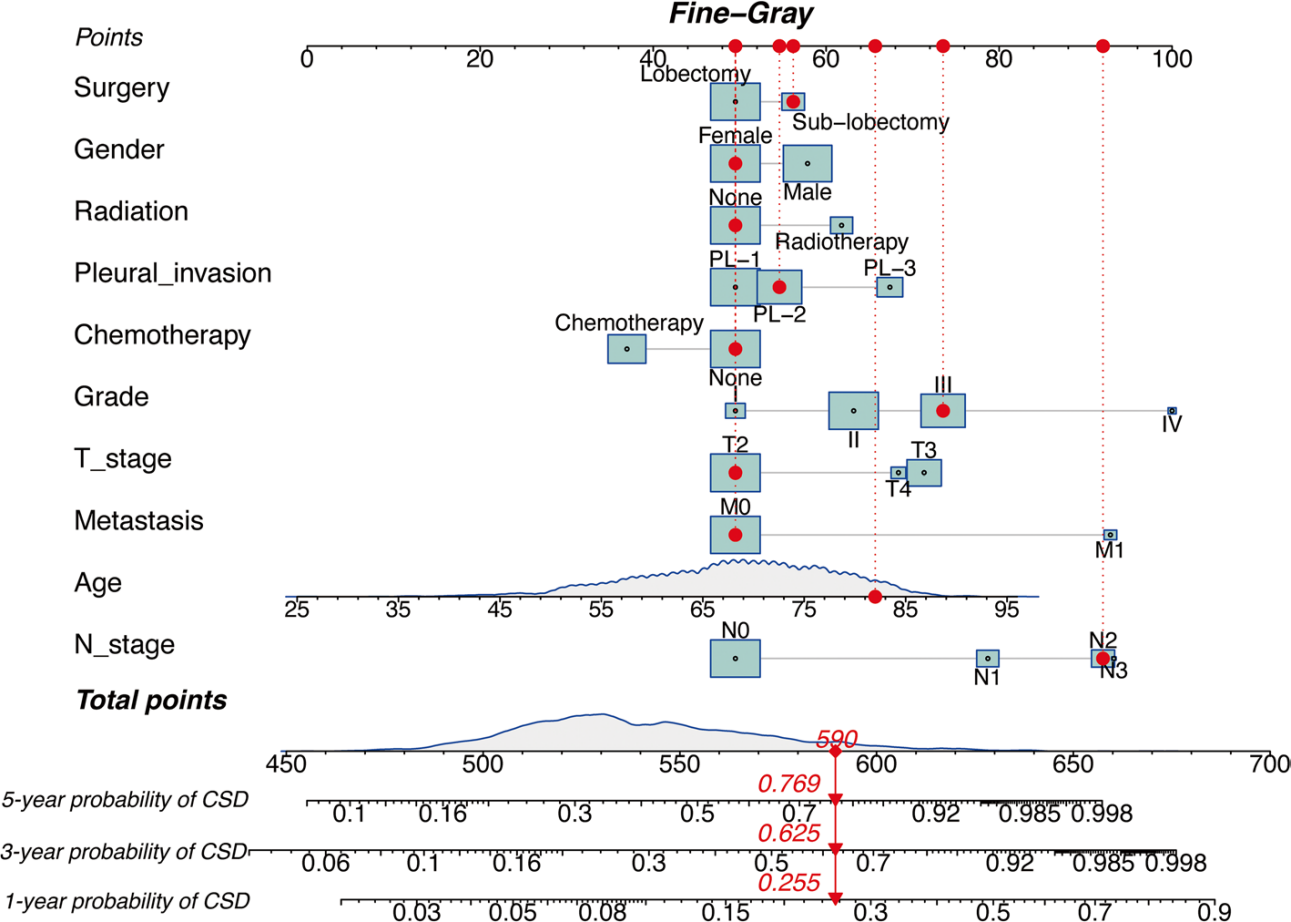
Nomogram based on the competing risk analysis to predict CSD probabilities at 1-, 3- and 5-year

A competing event nomogram was constructed to assess the 1-, 3-, and 5-year chances of CSD based on the variables from the multivariate analysis (Fig. 3). The total points were calculated by adding the scores for each patient’s prognostic characteristics, which clinicians can use to estimate the chance of CSD at different time points for specific patients.

**Fig. 4:**
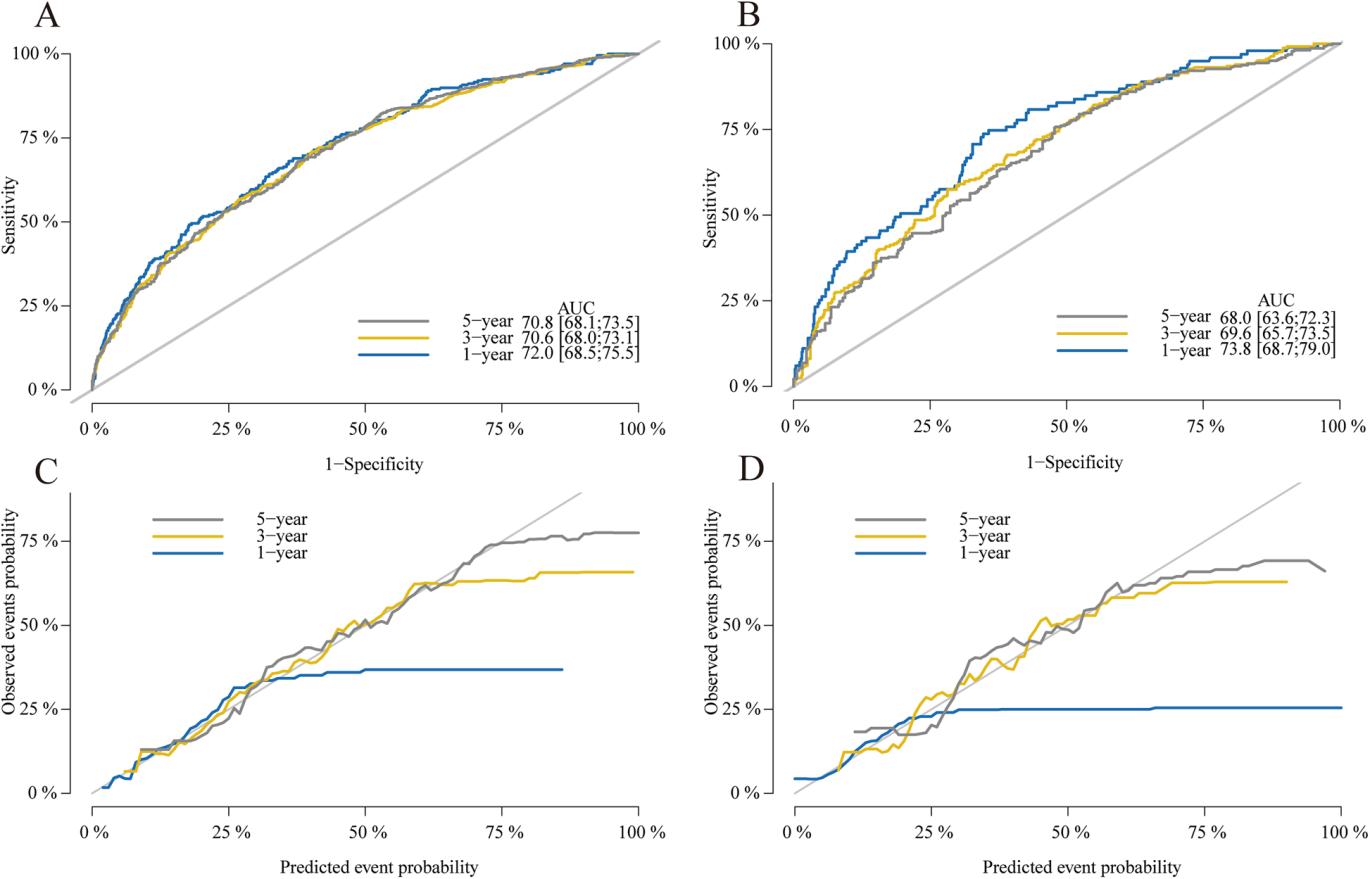
ROC curves at the 1-, 3-, and 5-year points in the training (**A**) and validation (**B**) cohort. Calibration curves at the 1-, 3-, and 5-year points in the training (**C**) and validation (**D**) cohort

The nomogram constructed using the training cohort was verified using the validation cohort. The 1-, 3-, 5-year AUC values were 0.720, 0.706, and 0.708 in the training cohort, and 0.738, 0.696, and 0.680 in the validation cohorts, respectively, which indicated good discrimination ability (Fig. 4A and B). We also used calibration plots to test the model’s prediction accuracy, which demonstrated relatively good consistency between the predicted and observed probabilities of CSD in both datasets (Fig. 4C and D). The above results illustrated the good predictive potential along with the high credibility of our nomogram.

## Discussion

The study demonstrated that in NSCLC patients diagnosed with PL, the cohort who underwent Lob presented lower CSD than the Sub-lob cohort. The use of a competing risk model could effectively eliminate the influence of death competition on cancer-specific survival, which indicated that these specific patients could obtain better survival from Lob. A competing event nomogram was constructed to individually predict the 1-, 3-, and 5-year chances of CSD among these patients. The model incorporating age, gender, grade, T stage, N stage, metastasis, pleural invasion, surgery, radiation, and chemotherapy presented favorable clinical applicability. To the best of our knowledge, this is the first study to compare Lob and Sub-lob in NSCLC patients with PL and construct a predictive nomogram.

PL is closely related to the aggressive biological behavior of pleural effusion, poor tumor differentiation, lymph node metastasis, postoperative recurrence, and even a dismal prognosis, which can directly affect the surgical strategies [4, 5]. Nowadays, Lob and Sub-lob are the most commonly adopted surgical methods for NSCLC [20]. However, there is no agreement regarding the better surgery type (Lob or Sub-lob) in NSCLC patients with PL. Several previous studies had compared the survival outcomes of Lob and Sub-lob in varying extent degrees of pleural infiltration. Choi et al. and Wo et al. demonstrated that among NSCLC patients with visceral PL, the cohort that underwent Lob resection presented an increased 5-year recurrence-free survival (RFS) rate and 5-year overall survival (OS) compared with the Sub-lob cohort [13, 21]. Likewise, Yu et al. indicated that Sub-lob presented inferior in contrast with Lob resection in long-term survival with visceral PL NSCLC patients [14]. Contrary to the studies mentioned above, Moon et al. investigated the surgical outcomes of 271 NSCLC patients with PL, indicating that the survival rate did not differ considerably depending on the extent of surgery [15]. The current study demonstrated that Lob was a favorable factor in low CSD before and after PSM compared with those treated with Sub-lob.

Several potential mechanisms may explain the benefit of survival outcomes from larger extent radical surgeries. Firstly, PL is correlated with a high incidence of lymph nodes metastasis [13, 22]. Kudo et al. discovered that the visceral pleura was densely packed with lymphatic vessels, with an interconnected network extending over the lung surface, The lymphatic vessels penetrated the lung parenchyma to connect bronchial lymph vessels with drainage to numerous hilar Lymph nodes [23]. Imai et al. indicated that lymphatic vessels beneath the pleura might flow directly into the mediastinum without going through the hilar Lymph nodes, resulting in skip N2 metastases [24]. Moreover, previous studies illustrated that more lymph nodes dissected in surgically resected NSCLC could improve the survival rate [25, 26]. Compared with Sub-lob, Lob, which tends to perform more comprehensive lymph nodes excision and obtain R0 resection, is associated with a better prognosis among NSCLC patients with PL [13, 22]. Secondly, Sub-lob can easily impair the lymph nodes’ integrity and disrupt the drainage system, resulting in decreased lymphatic fluid release during segmental lymph nodes dissection. Finally, regardless of how carefully a Sub-lob of NSCLC is performed, the possibility of cancer cells at the surgical margin remains, which is associated with locoregional recurrence and a poor prognosis [27, 28].

Interestingly, in the subgroups analyses, only the patients in the PL-2 cohort presented significantly different survival outcomes, indicating lower CSD in the cases that underwent Lob. In surgically resected NSCLC, the poor prognostic impact of PL has been clearly outlined. Nevertheless, the prognostic significance of the PL depth, especially in PL-1 and PL-2, was still under debate. Some [29–33] but not all studies [34–36] confirmed that resected NSCLC patients with PL-2 had a significantly worse prognosis, frequent recurrence [31], and pleural dissemination [33] contrast with PL-1. A high level of PL was commonly associated with increasingly aggressive biological characteristics. Kondo et al. reported that the pleural lavage cytology was positive in 13 of 96 (14%) and 15 of 41 (37%) patients in the PL-1 and PL-2 groups [37]. However, the above studies did not carry out the prognostic outcomes of PL depth between different surgical operations. As previous research mentioned, Lob could achieve more extensive lymphatic clearance and reduce the possibility of cancer cells remnant. And PL-2 patients tended to show the characteristics of local lymph node metastasis and recurrence. We supported the view that in NSCLC patients, PL depth should be considered in selecting the optimal surgery type. Conventional preoperative diagnostic methods were limited in diagnosing PL. However, with the exploration of new technology such as circulating tumor cells combined with CT features, and artificial-intelligence CT texture features, a more accurate preoperative judgment of PL may be realized [38, 39].

This study comprehensively compared the CSD of Lob and Sub-lob in the NSCLC patients with PL. Meanwhile, a relatively accurate and discriminating nomogram was developed and internally validated. Based on the competing risk analysis, the model incorporated age, gender, grade, T stage, N stage, metastasis, pleural invasion, surgery, radiation, and chemotherapy. It was reported that age and grade were closely related to prognosis in NSCLC [32, 40]. And the model showed that patients who underwent Sub-lob were associated with an increased risk for CSD. The reasons have been discussed in the previous paragraphs. Higher extent of PL was also correlated with more CSD probability. It was reported that malignant tumor cells have increasingly aggressive and progressive biological characteristics with the increasing level of PL and contribute to adverse outcomes for the patients [32]. The advantage of nomogram over standard multivariate regression model was providing the individual probability of 1-, 3-, 5-year CSD instead of a relative risk concept. The listed factors can be easily obtained from clinical and pathological data. Besides, our nomogram presented favorable potential clinical applicability and could contribute to patient counseling, follow-up scheduling, and treatment selection.

However, several limitations that existed in the current study should raise attention. Firstly, the SEER data repository lacked some pivotal factors tied to prognosis, including smoking history, comorbidities, and genetic records of patients. Secondly, as a retrospective analysis, although PSM was used to minimize the heterogeneity between the groups, selection bias was inevitably brought in. Despite these limitations, the large cases could provide novel insights into the surgical treatment in PL NSCLC patients.

## Conclusion

Our findings supported that Lob should be considered the preferred surgery in contrast with Sub-lob for NSCLC patients with PL. A prognostic nomogram was constructed and validated to predict the individualized probability of CSD at 1-, 3- and 5-year, which presented excellent prediction ability for these patients. External and prospective validation was required for widely applying.

## Supplementary Information

**Additional file 1 **: **Figure S1.** The mean difference between the two cohorts.

**Additional file 2 **: **Figure S2.** Cumulative incidence curves for the NSCLC patients with PL in overall cases and different subgroups before PSM. Overall patients (A), T2 (B), T3 (C), T4 (D), N0 (E), N1 (F), N2 (G), M0 (H), M1 (I), adenocarcinoma (J), squamous cell carcinoma (K), PL-1 (L), PL-2 (M) and PL-3 (N) cohorts.

**Additional file 3 **: **Table S1.** The results of the multivariate subdistribution hazards model on OCD before and after PSM.

**Additional file 4 **: **Table S2.** The basic characteristics in the training and validation cohorts.

## Data Availability

The datasets created and analyzed during the current investigation are accessible from the corresponding authors and the SEER database (https://seer.cancer.gov/).

## Abbreviations

PL: Pleural invasion
NSCLC: Non-small cell lung cancer
CSD: Cancer-specific death
PSM: Propensity score matching
Lob: Lobectomy
Sub-lob: Sub-lobectomy
SEER: Surveillance, Epidemiology, and End Results
AUC: Area under the receiver operating characteristic curve
OCD: Other causes death
CIF: Cumulative incidence function
SD: Standardized difference
ROC: Receiver operating characteristic

## References

[1] Siegel R, Miller K, Jemal A (2018). Cancer statistics, 2018. CA Cancer J Clin.

[2] Jemal A, Center M, DeSantis C, Ward E (2010). Global patterns of cancer incidence and mortality rates and trends. Cancer Epidemiol Biomark Prev.

[3] Morgensztern D, Ng S, Gao F, Govindan R (2010). Trends in stage distribution for patients with non-small cell lung cancer: a National Cancer Database survey. J Thorac Oncol.

[4] Shimizu K, Yoshida J, Nagai K, Nishimura M, Ishii G, Morishita Y (2005). Visceral pleural invasion is an invasive and aggressive indicator of non-small cell lung cancer. J Thorac Cardiovasc Surg.

[5] Manac'h D, Riquet M, Medioni J, Le Pimpec-Barthes F, Dujon A, Danel C (2001). Visceral pleura invasion by non-small cell lung cancer: an underrated bad prognostic factor. Ann Thorac Surg.

[6] Deng H, Li G, Luo J, Alai G, Zhuo Z, Lin Y (2018). Novel biologic factors correlated to visceral pleural invasion in early-stage non-small cell lung cancer less than 3 cm. J Thorac Dis.

[7] Agalioti T, Giannou A, Stathopoulos G (2015). Pleural involvement in lung cancer. J Thorac Dis.

[8] De Giglio A, Di Federico A, Gelsomino F, Ardizzoni A (2021). Prognostic relevance of pleural invasion for resected NSCLC patients undergoing adjuvant treatments: A propensity score-matched analysis of SEER database. Lung Cancer.

[9] Zhang X, Xie J, Hu S, Peng W, Xu B, Li Y (2021). Prognostic value of visceral pleural invasion in the stage pT1-2N2M0 non-small cell lung cancer: A study based on the SEER registry. Curr Probl Cancer.

[10] Cao C, Gupta S, Chandrakumar D, Tian D, Black D, Yan T (2014). Meta-analysis of intentional sublobar resections versus lobectomy for early stage non-small cell lung cancer. Ann Cardiothorac Surg.

[11] Gupta S, Yan T, Tian D (2015). Could less be more?-A systematic review and meta-analysis of sublobar resections versus lobectomy for non-small cell lung cancer according to patient selection. Lung Cancer.

[12] Uramoto H, Tanaka F (2014). Recurrence after surgery in patients with NSCLC. Transl Lung Cancer Res.

[13] Wo Y, Zhao Y, Qiu T, Li S, Wang Y, Lu T (2019). Impact of visceral pleural invasion on the association of extent of lymphadenectomy and survival in stage I non: mall cell lung cancer. Cancer Med.

[14] Yu Y, Huang R, Wang P, Wang S, Ling X, Zhang P (2020). Sublobectomy versus lobectomy for long-term survival outcomes of early-stage non-small cell lung cancer with a tumor size </=2 cm accompanied by visceral pleural invasion: a SEER population-based study. J Thorac Dis.

[15] Moon Y, Lee KY, Park JK (2017). Prognosis after sublobar resection of small-sized non-small cell lung cancer with visceral pleural or lymphovascular invasion. World J Surg.

[16] Filleron T, Laplanche A, Boher JM, Kramar A (2010). An R function to non-parametric and piecewise analysis of competing risks survival data. Comput Methods Prog Biomed.

[17] Austin P (2011). An introduction to propensity score methods for reducing the effects of confounding in observational studies. Multivar Behav Res.

[18] Austin P (2009). Balance diagnostics for comparing the distribution of baseline covariates between treatment groups in propensity-score matched samples. Stat Med.

[19] Zhang Z, Geskus RB, Kattan MW, Zhang H, Liu T (2017). Nomogram for survival analysis in the presence of competing risks. Ann Transl Med.

[20] Ginsberg RJ, Rubinstein LV (1995). Randomized trial of lobectomy versus limited resection for T1 N0 non-small cell lung cancer. Lung Cancer Study Group. Ann Thorac Surg.

[21] Choi SY, Moon MH, Moon Y (2020). The prognosis of small-sized non-small cell lung cancer with visceral pleural invasion after sublobar resection. Transl Cancer Res.

[22] Fujimoto T, Cassivi S, Yang P, Barnes S, Nichols F, Deschamps C (2006). Completely resected N1 non-small cell lung cancer: factors affecting recurrence and long-term survival. J Thorac Cardiovasc Surg.

[23] Kudo Y, Saji H, Shimada Y, Nomura M, Matsubayashi J, Nagao T (2012). Impact of visceral pleural invasion on the survival of patients with non-small cell lung cancer. Lung Cancer.

[24] Imai K, Minamiya Y, Saito H, Nakagawa T, Ito … M. (2013). Detection of pleural lymph flow using indocyanine green fluorescence imaging in non-small cell lung cancer surgery: a preliminary study. Surg Today.

[25] Liang W, He J, Shen Y, et al. Impact of examined lymph node count on precise staging and long-term survival of resected non-small-cell lung cancer: a population study of the US SEER database and a Chinese multi-institutional registry. J Clin Oncol. 2017;35(11):1162–70. 10.1200/JCO.2016.67.5140 PMC5455598 28029318

[26] Samayoa AX, Pezzi TA, Pezzi CM, Gay EG, Asai M, Kulkarni N, et al. Rationale for a minimum number of lymph nodes removed with non-small cell lung cancer resection: correlating the number of nodes removed with survival in 98,970 Patients. Ann Surg Oncol. 2016;23(5):S1005-11. 10.1245/s10434-016-5509-4 27531307

[27] Sawabata N (2013). Locoregional recurrence after pulmonary sublobar resection of non-small cell lung cancer: can it be reduced by considering cancer cells at the surgical margin?. Gen Thorac Cardiovasc Surg.

[28] Sawabata N, Maeda H, Matsumura A, Ohta M, Okumura M (2012). Clinical implications of the margin cytology findings and margin/tumor size ratio in patients who underwent pulmonary excision for peripheral non-small cell lung cancer. Surg Today.

[29] Eberhardt W, Alan M, John C, Haruhiko K, Tae KY, Andrew T, et al. The IASLC lung cancer staging project: proposals for the revision of the M descriptors in the forthcoming eighth edition of the TNM classification of lung cancer. J Thorac Oncol. 2016;10(11);1515-22. 10.1097/JTO.0000000000000673 26536193

[30] Akikazu K, Junji Y, Etsuo M (2013). Visceral pleural invasion classification in non–small-cell lung cancer in the 7th edition of the tumor, node, metastasis classification for lung cancer: validation analysis based on a large-scale nationwide database. J Thorac Oncol.

[31] Hung JJ, Jeng WJ, Hsu WH, Chou TY, Lin SF, Wu YC (2012). Prognostic significance of the extent of visceral pleural invasion in completely resected node-negative non-small cell lung cancer. Chest.

[32] Wang T, Zhou C, Zhou Q (2017). Extent of visceral pleural invasion affects prognosis of resected non-small cell lung cancer: a meta-analysis. Sci Rep.

[33] Liang RB, Li P, Li BT, Jin JT, Rusch VW, Jones DR (2021). Modification of pathologic T classification for non-small cell lung cancer with visceral pleural invasion: data From 1,055 Cases of Cancers </= 3 cm. Chest.

[34] Kawase A, Yoshida J, Ishii G, Hishida T, Nishimura M, Nagai K (2010). Visceral pleural invasion classification in non-small cell lung cancer. J Thorac Oncol.

[35] Shim HS, Park IK, Lee CY, Chung KY (2009). Prognostic significance of visceral pleural invasion in the forthcoming (seventh) edition of TNM classification for lung cancer. Lung Cancer.

[36] Shimizu K, Yoshida J, Nagai K, Nishimura M, Yokose T, Ishii G (2004). Visceral pleural invasion classification in non–small cell lung cancer: a proposal on the basis of outcome assessment. J Thorac Cardiovasc Surg.

[37] Kondo H (1993). Prognostic significance of pleural lavage cytology immediately after thoracotomy in patients with lung cancer. J Thorac Cardiovasc Surg.

[38] Shi J, Li F, Yang F, Dong Z, Jiang Y, Nachira D (2021). The combination of computed tomography features and circulating tumor cells increases the surgical prediction of visceral pleural invasion in clinical T1N0M0 lung adenocarcinoma. Transl Lung Cancer Res.

[39] Zuo Z, Li Y, Peng K, Li X, Tan Q, Mo Y (2022). CT texture analysis-based nomogram for the preoperative prediction of visceral pleural invasion in cT1N0M0 lung adenocarcinoma: an external validation cohort study. Clin Radiol.

[40] Zhai X, Guo Y, Qian X (2020). Combination of fluorine-18 fluorodeoxyglucose positron-emission tomography/computed tomography (^18^F-FDG PET/CT) and tumor markers to diagnose lymph node metastasis in non-small cell lung cancer (NSCLC): a retrospective and prospective study. Med Sci Monit.

