# Supplementary figures and images for: Is lobectomy superior to sub-lobectomy in non-small cell lung cancer with pleural invasion? A population-based competing risk analysis
Source: BMC Cancer. 2022 May 13;22:541. doi: 10.1186/s12885-022-09634-w (PMC9102677; doi:10.1186/s12885-022-09634-w)

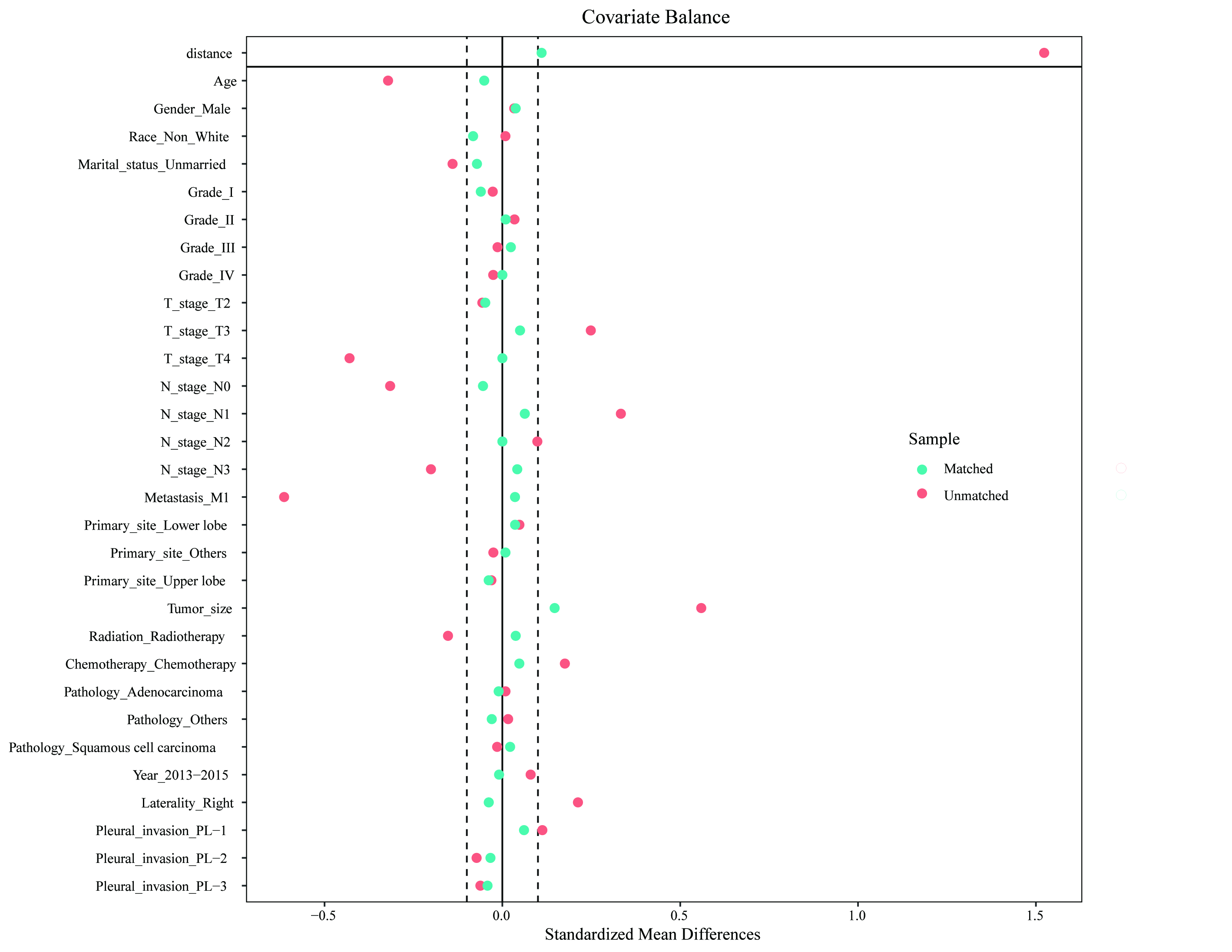

Supplement: Supplementary file 1 — Additional file 1 : Figure S1. The mean difference between the two cohorts. [file 12885_2022_9634_MOESM1_ESM.tif]

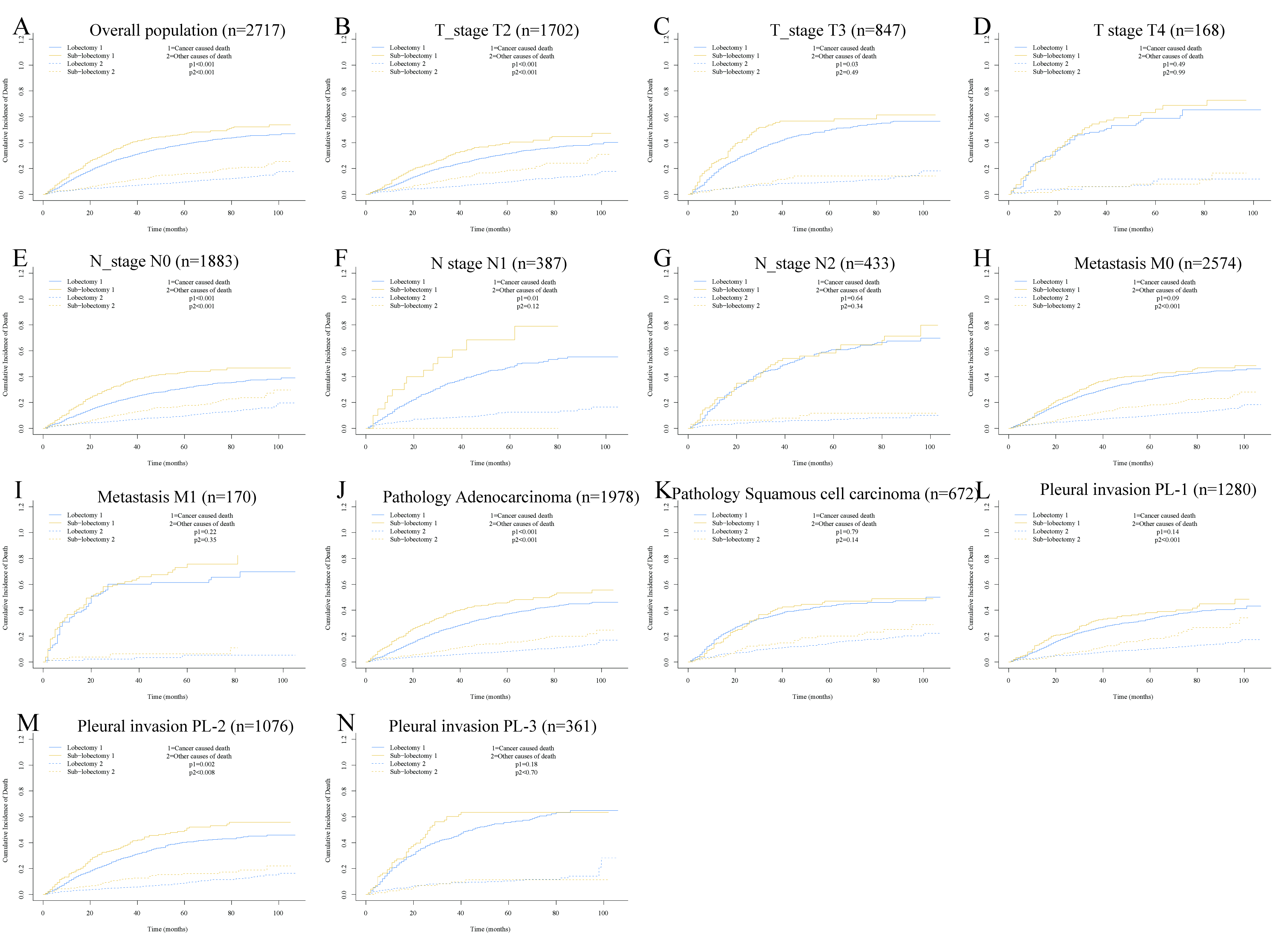

Supplement: Supplementary file 2 — Additional file 2 : Figure S2. Cumulative incidence curves for the NSCLC patients with PL in overall cases and different subgroups before PSM. Overall patients (A), T2 (B), T3 (C), T4 (D), N0 (E), N1 (F), N2 (G), M0 (H), M1 (I), adenocarcinoma (J), squamous cell carcinoma (K), PL-1 (L), PL-2 (M) and PL-3 (N) cohorts. [file 12885_2022_9634_MOESM2_ESM.tif]
