# Supplementary material for: Is lobectomy superior to sub-lobectomy in non-small cell lung cancer with pleural invasion? A population-based competing risk analysis
Source: BMC Cancer. 2022 May 13;22:541. doi: 10.1186/s12885-022-09634-w (PMC9102677; doi:10.1186/s12885-022-09634-w)
Supplement: Supplementary file 3 — Additional file 3 : Table S1. The results of the multivariate subdistribution hazards model on OCD before and after PSM. [file 12885_2022_9634_MOESM3_ESM.docx]

| Table S1. The results of the multivariate subdistribution hazards model on OCD before and after PSM | | | | | | |
| --- | --- | --- | --- | --- | --- | --- |
| Characteristics | Before PSM | | | After PSM | | |
|  | HR | 95%CI | P value | HR | 95%CI | P value |
| Surgery |  |  |  |  |  |  |
| Lobectomy | Reference |  |  | Reference |  |  |
| Sub-lobectomy | 1.41 | 1.08-1.84 | 0.013 | 1.37 | 0.96-1.95 | 0.078 |
| Age | 1.03 | 1.02-1.05 | <0.001 | 1.04 | 1.02-1.06 | <0.001 |
| Gender |  |  |  |  |  |  |
| Female | Reference |  |  | Reference |  |  |
| Male | 1.49 | 1.2-1.87 | <0.001 | 1.39 | 0.97-2.01 | 0.074 |
| Race |  |  |  |  |  |  |
| White | Reference |  |  | Reference |  |  |
| Non-White | 0.64 | 0.46-0.88 | 0.007 | 0.57 | 0.32-1.01 | 0.052 |
| Marital status |  |  |  |  |  |  |
| Married | Reference |  |  | Reference |  |  |
| Unmarried | 1.44 | 1.15-1.79 | 0.001 | 1.29 | 0.91-1.83 | 0.161 |
| Grade |  |  |  |  |  |  |
| I | Reference |  |  | Reference |  |  |
| II | 1.32 | 0.84-2.08 | 0.221 | 1.11 | 0.57-2.14 | 0.763 |
| III | 1.05 | 0.65-1.69 | 0.840 | 0.91 | 0.45-1.85 | 0.833 |
| IV | 0.31 | 0.04-2.74 | 0.296 | 0 | 0-0 | <0.001 |
| T stage |  |  |  |  |  |  |
| T2 | Reference |  |  | Reference |  |  |
| T3 | 1.22 | 0.88-1.68 | 0.244 | 0.79 | 0.38-1.63 | 0.521 |
| T4 | 1.13 | 0.65-1.97 | 0.656 | 0.45 | 0.16-1.29 | 0.145 |
| N stage |  |  |  |  |  |  |
| N0 | Reference |  |  | Reference |  |  |
| N1 | 1.28 | 0.9-1.83 | 0.177 | 0.69 | 0.23-2.07 | 0.511 |
| N2 | 0.91 | 0.61-1.36 | 0.653 | 0.87 | 0.43-1.75 | 0.695 |
| N3 | 1.28 | 0.18-9.1 | 0.810 | 0 | 0-0 | <0.001 |
| Metastasis |  |  |  |  |  |  |
| M0 | Reference |  |  | Reference |  |  |
| M1 | 0.54 | 0.27-1.07 | 0.075 | 0.32 | 0.09-1.12 | 0.074 |
| Pathology |  |  |  |  |  |  |
| Adenocarcinoma | Reference |  |  | Reference |  |  |
| Others | 0.67 | 0.19-2.3 | 0.521 | 0 | 0-0 | <0.001 |
| Squamous cell carcinoma | 1.38 | 1.08-1.77 | 0.011 | 1.25 | 0.84-1.86 | 0.271 |
| Pleural invasion |  |  |  |  |  |  |
| PL-1 | Reference |  |  | Reference |  |  |
| PL-2 | 0.86 | 0.68-1.1 | 0.235 | 0.75 | 0.52-1.1 | 0.145 |
| PL-3 | 0.99 | 0.65-1.49 | 0.956 | 1.08 | 0.47-2.5 | 0.853 |
| Primary site |  |  |  |  |  |  |
| Lower lobe | Reference |  |  | Reference |  |  |
| Others | 0.92 | 0.59-1.44 | 0.722 | 1.08 | 0.54-2.15 | 0.833 |
| Upper lobe | 0.9 | 0.71-1.15 | 0.416 | 0.96 | 0.63-1.44 | 0.836 |
| Laterality |  |  |  |  |  |  |
| Left | Reference |  |  | Reference |  |  |
| Right | 0.93 | 0.74-1.18 | 0.563 | 1.03 | 0.73-1.47 | 0.861 |
| Tumor size | 0.92 | 0.86-0.98 | 0.017 | 1 | 0.92-1.09 | 0.997 |
| Radiation |  |  |  |  |  |  |
| None | Reference |  |  | Reference |  |  |
| Radiotherapy | 0.95 | 0.67-1.34 | 0.765 | 0.99 | 0.6-1.64 | 0.973 |
| Chemotherapy |  |  |  |  |  |  |
| None | Reference |  |  | Reference |  |  |
| Chemotherapy | 0.55 | 0.4-0.76 | <0.001 | 0.88 | 0.47-1.63 | 0.680 |
| HR: Hazard ratio |  |  |  |  |  |  |
