# Supplementary material for: Is lobectomy superior to sub-lobectomy in non-small cell lung cancer with pleural invasion? A population-based competing risk analysis
Source: BMC Cancer. 2022 May 13;22:541. doi: 10.1186/s12885-022-09634-w (PMC9102677; doi:10.1186/s12885-022-09634-w)
Supplement: Supplementary file 4 — Additional file 4 : Table S2. The basic characteristics in the training and validation cohorts. [file 12885_2022_9634_MOESM4_ESM.docx]

| Table S2. The basic characteristics in the training and validation cohorts | | | |
| --- | --- | --- | --- |
| Charactersitics | Training cohort | Validation cohort | P value |
|  | N=1902 | N=815 |  |
| Year at diagnosis |  |  | 0.433 |
| 2010-2012 | 901 (47.4%) | 372 (45.6%) |  |
| 2013-2015 | 1001 (52.6%) | 443 (54.4%) |  |
| Age | 69.0 (13.1) | 68.0 (14.2) | 0.731 |
| Gender |  |  | 0.589 |
| Female | 973 (51.2%) | 407 (49.9%) |  |
| Male | 929 (48.8%) | 408 (50.1%) |  |
| Race |  |  | 0.521 |
| White | 1523 (80.1%) | 662 (81.2%) |  |
| Non-White | 379 (19.9%) | 153 (18.8%) |  |
| Marital status: |  |  | 0.239 |
| Married | 1133 (59.6%) | 465 (57.1%) |  |
| Unmarried | 769 (40.4%) | 350 (42.9%) |  |
| Grade: |  |  | 0.848 |
| I | 151 (7.9%) | 67 (8.2%) |  |
| II | 955 (50.2%) | 394 (48.3%) |  |
| III | 770 (40.5%) | 342 (42.0%) |  |
| IV | 26 (1.4%) | 12 (1.5%) |  |
| T stage: |  |  | 0.101 |
| T2 | 1207 (63.5%) | 495 (60.7%) |  |
| T3 | 589 (31.0%) | 258 (31.7%) |  |
| T4 | 106 (5.6%) | 62 (7.6%) |  |
| N stage: |  |  | 0.404 |
| N0 | 1336 (70.2%) | 547 (67.1%) |  |
| N1 | 265 (13.9%) | 122 (15.0%) |  |
| N2 | 291 (15.3%) | 142 (17.4%) |  |
| N3 | 10 (0.5%) | 4 (0.5%) |  |
| Metastasis: |  |  | 0.665 |
| M0 | 1786 (93.9%) | 761 (93.4%) |  |
| M1 | 116 (6.1%) | 54 (6.6%) |  |
| Pathology: |  |  | 0.852 |
| Adenocarcinoma | 1383 (72.7%) | 595 (73.0%) |  |
| Others | 49 (2.6%) | 18 (2.2%) |  |
| Squamous cell carcinoma | 470 (24.7%) | 202 (24.8%) |  |
| Pleural invasion: |  |  | 0.461 |
| PL-1 | 910 (47.8%) | 370 (45.4%) |  |
| PL-2 | 746 (39.2%) | 330 (40.5%) |  |
| PL-3 | 246 (12.9%) | 115 (14.1%) |  |
| Primary site: |  |  | 0.072 |
| Lower lobe | 577 (30.3%) | 225 (27.6%) |  |
| Others | 163 (8.6%) | 56 (6.9%) |  |
| Upper lobe | 1162 (61.1%) | 534 (65.5%) |  |
| Laterality: |  |  | 0.601 |
| Left | 769 (40.4%) | 339 (41.6%) |  |
| Right | 1133 (59.6%) | 476 (58.4%) |  |
| Tumor size | 3.0 (0.9) | 3.0 (1.1) | 0.539 |
| Surgery: |  |  | 0.092 |
| Lobectomy | 1577 (82.9%) | 653 (80.1%) |  |
| Sub-lobectomy | 325 (17.1%) | 162 (19.9%) |  |
| Radiation: |  |  | 0.024 |
| None | 1583 (83.2%) | 648 (79.5%) |  |
| Radiotherapy | 319 (16.8%) | 167 (20.5%) |  |
| Chemotherapy: |  |  | 0.023 |
| None | 1189 (62.5%) | 471 (57.8%) |  |
| Chemotherapy | 713 (37.5%) | 344 (42.2%) |  |
